# The joint effect of unemployment and cynical hostility on all-cause mortality: results from a prospective cohort study

**DOI:** 10.1186/s12889-019-6622-7

**Published:** 2019-03-12

**Authors:** Margit Kriegbaum, Rikke Lund, Lone Schmidt, Naja Hulvej Rod, Ulla Christensen

**Affiliations:** Department of Public Health, University of Copenhagen, Oester Farimagsgade 5, 1014 Copenhagen K, Denmark

**Keywords:** Hostility, Unemployment, Joint effect, Mortality, Psychosocial vulnerability

## Abstract

**Background:**

It is hypothesised that hostility accentuates the association between stressful conditions and health. This study aims to test this hypothesis by analysing the joint effect of unemployment and hostility on all-cause mortality among men and women.

**Methods:**

The population was 3677 men and 4138 women from the Danish workforce who participated in a survey in 2000. The joint exposure variable was defined as 1) employed, not hostile, 2) unemployed, not hostile, 3) hostile and employed, 4) unemployed and hostile. Outcome was defined as all-cause mortality between 2000 and 2014. Data was analysed with Cox proportional hazards models with age as the underlying time scale. The interaction between unemployment and hostility was studied using the synergy index.

**Results:**

Compared to employed non-hostile men, men who were both hostile and unemployed were at markedly higher risk of premature death with a hazard ratio (HR) of 3.19 (95% CI 2.22–4.69). A similar picture was found for hostile and unemployed women, with a HR of 1.97 (95% CI 1.24–3.12). However, the mortality in men and women exposed to both did not exceed what was expected from the combination of their individual effects. Hence, we did not find that hostility enhances the association between unemployment and all-cause mortality.

**Conclusion:**

Men and women exposed to both unemployment and hostility were at markedly high risk of premature mortality. However, this study did not support the hypothesis that the deleterious health effect of the combination of unemployment and hostility exceeds their individual effects.

**Electronic supplementary material:**

The online version of this article (10.1186/s12889-019-6622-7) contains supplementary material, which is available to authorized users.

## Background

Several studies have shown unemployment to be associated with both psychological distress and all-cause and cause-specific mortality in men and women [1–8]. One expression of psychological distress is hostility, a multifaceted construct defined as an enduring, negative attitude towards others involving cognitive, affective, and behavioural components [9]. The relationship between the dimensions of hostility and negative health outcomes is well established. Hostility has been associated with higher symptom load and poorer self-rated health [10, 11], with adverse health-related behaviours [9], depression [9, 12, 13], incident cardiovascular disease (CVD) [12–15], and premature death [16–20].

Hostility has been related to socioeconomic position and employment status. Hostility was, for example, associated with low educational attainment and low occupational social class in a national sample of American adults aged 18 to 90 years [21]. Also, in the British Whitehall II study, higher scores of hostility have been related to lower occupational grade [22–24]. A similar picture is seen in middle-aged Danish men and women, where the prevalence of high hostility among men and women receiving benefits, including unemployment, was nearly four times as high as those in the high-occupational social class [10]. In these studies, hostility has mainly reflected a sceptical approach to other peoples’ sincerity, motives, and beliefs and showing a basic mistrust of others.

Little is known about the health consequences of being exposed to both unemployment and hostility. The psychosocial vulnerability model has been proposed as a hypothetical model for the association between hostility and health [11]. According to this model, hostile persons are less likely to engage in social interaction and have a lower stress-buffering potential from interpersonal support, which may predict worse health outcomes when exposed to a stressor such as unemployment [11, 25]. Kivimäki and co-authors have explained the psychosocial vulnerability model as an interaction between hostility and adverse social conditions that leads to an increase in negative health outcomes among hostile individuals because of their lower capacity to benefit from existing psychosocial resources [11]. Few previous studies have investigated the effect modification of hostility on the relationship between social conditions and health, and only two studies from Finland [26, 27] have been aimed directly at testing the psychosocial vulnerability hypothesis. The psychosocial vulnerability hypothesis was only partly supported in these studies as the moderating effect of hostility seemed to be gender-specific. In one study, there was no interaction between hostility and unemployment on self-rated health among women, but interaction was found among men, that is, hostility accentuated the effect of unemployment on self-rated health in men [26]. In the other study conducted over five years with economic decline, hostile women had a higher risk of sickness absence after exposure to stressors such as organisational downsising, job insecurity, and a stressful psychosocial work environment. This increased vulnerability was not seen among hostile men exposed to the same stressors [27].

Methodologically, these studies investigated if the association between social factors and health differed in strata of hostile vs. non-hostile individuals (i.e., effect modification). We suggest that a measure of interaction, which aims to study effects that are due to interaction, that is, cases that would not have occurred if it was not for the presence of both risk factors. This is relevant in public health because the blocking of pathways to poor health that involves synergistic effects can be powerful because the absolute number of cases that can be prevented is higher than the number of cases from individual risk factors. If there is a synergistic effect of being exposed to both unemployment and hostility, interventions directed at this group would be highly efficient.

While previous studies to some extent support the vulnerability hypothesis, no previous studies have, to our knowledge, focused on how the joint effect of unemployment and hostility affects all-cause mortality.

We hypothesised that the joint effects of unemployment and hostility on mortality exceed their individual effects. We aim to test this hypothesis by evaluating the joint effect of unemployment and hostility on all-cause mortality among a large sample of Danish men and women, followed prospectively in a nationwide mortality register.

## Methods

### Data and participants

The Danish Longitudinal Study on Work, Unemployment, and Health is a prospective population-based study with a baseline postal survey carried out in 2000. The baseline survey was based on a stratified random sample consisting of two population groups: 1) a group of individuals 40 and 50 years old by 1 October, 1999, (response rate 69%, *n* = 7588), and 2) a group of 37–56-year-old individuals who had been unemployed at least 70% of the time during the period 1 October, 1996 to 1 October, 1999, (response rate 57%, *n* = 2287). The latter group was included to ensure a sufficient number of unemployed which was one of the main interests of the study. In 1999, the unemployment rate was low with 6% unemployed among both the 40- and 50-year-old. Both samples have initially been drawn from the ‘AKF Longitudinal Register’, which is maintained by the National Centre for Social Science Research (previously AKF) and comprises all Danes aged 15 years or older. Data on non-participation was derived from the registers and showed that non-participants were more often men, non-native-born Danes, persons living on transfer income, and persons with low educational attainment (non-trained or semi-skilled). Out of 9875 participants in the survey, we were able to link 9870 to register data. The Cause of Death Registry, The Danish National Population Register, The National Patient Registry, and the National Drug Prescription Registry were used to identify mortality and covariates. For the present study, an inclusion criterion was those in the workforce. The workforce is defined as those who are working, plus those who are available for work. From the survey, we selected individuals who were working or unemployed at the time of the survey (8733). Of these, 223 (3%) had missing information on hostility, and 100 (1%) had missing information on education. The final study population was 8426. The details of the population selection are available in Additional file 1.

### Assessment of unemployment and hostility

Information on employment status was based on self-reported information on current labour market participation in 2000 and grouped as ‘employed’ or ‘unemployed’. Hostility was measured by the eight-item Cynical Distrust Scale, derived from the Cook-Medley Hostility Scale, originally based on the items from the Minnesota Teacher Attitude Inventory [28]. This scale measures a sceptical approach to other peoples’ sincerity, motives, and beliefs and the showing of a basic mistrust of others. This cynical mistrust dimension of hostility has been most solidly associated with measures of low social position and, consequently, we found it the most relevant measure to include in this specific study. The Cynical Distrust Scale that measured the cognitive component of hostility was factor-analytically derived from the Cook-Medley Scale by Greenglass and Julkunen [29, 30]. In two separate samples of Canadian and Finnish students, they demonstrated that the shorter Cynical Distrust Scale is a sufficiently valid, reliable, and specific measure of cynicism and distrust when compared to the full Cook-Medley Scale [29, 30]. In the present study, we used the following eight items as presented in English by Everson et al. [17]:

1) I think most people would lie to get ahead. 2) Most people inwardly dislike putting themselves out to help other people. 3) Most people make friends because friends are likely to be useful to them. 4) It is safer to trust nobody. 5) No one cares much what happens to you. 6) Most people are honest chiefly through fear of being caught. 7) I commonly wonder what hidden reasons another person may have for doing something nice to me. 8) Most people will use somewhat unfair means to gain profit or an advantage rather than lose it.

We translated the items into Danish and adjusted the wording after a back-translation into English. The Danish version was tested in a large-scale pilot study of the entire questionnaire among a study population (*n* = 993) drawn from the same sampling frame as the main survey [10]. This work was led by the last author of this manuscript. Response options were: completely agree, somewhat agree, somewhat disagree, completely disagree. In the analyses, the items were summed to obtain a Cynical Distrust Scale score with a range of 0–24. The internal reliability was tested by Cronbach’s alpha, which at 0.89 was satisfactory. In the literature, there is no established threshold for dichotomisation of the Cynical Distrust Scale. To define the cut point for dichotomy, we divided the scale into quartiles as has been applied in the studies by Everson et al. and Stamatakis et al. [17, 31], and we used separate cut points for men (scoring 9+) and women (scoring 7+).

To study the joint exposure to hostility and unemployment, a new composite variable with four categories was defined: 1) unexposed (reference), 2) unemployed, but not hostile, 3) hostile but not unemployed, 4) unemployed and hostile. The joint reference category allows us to compare each combination of hostility and unemployment level according to the same baseline hazard.

### Assessment of covariates

Potential confounders for the analyses were identified based on prior knowledge and the method of directed acyclic graphs (DAG) [32]. DAGs provide a graphical tool for identification and selection of relevant confounders in epidemiological studies based on prior substance knowledge. The DAG and overview of included variables are available in Additional file 2. Cohabitation status retrieved from the population registry in 1999 was defined as living with vs. without a partner. Educational attainment was based on self-reported information on the type of vocational training following primary school and categorised into no education (no training—only primary school) or some education (any level beyond primary school). Information on alcohol abuse and depression was retrieved from The National Patient Registry 1980–1999 and the National Drug Prescription Registry 1995–1999, which contains information about outpatient prescription drug use. We used the International Classification of Disease (ICD) to identify diagnoses. We included admissions for alcohol, cirrhosis of the liver (571.09; 571.19 ICD 8 /I85.0-I85.9 ICD 10), and Oesophageal varices (456.09; 456.19 ICD 8). Further, a prescription for alcohol dependence (ATC code N07BB) was used to identify alcohol abuse. Depression was defined as at admissions with bipolar depression, depression, and recurrent depression (ICD8: 296, 298.09, 298.19; ICD-10: F30-F33.9) and/or prescriptions with antidepressants (ATC code N06A). The variable for alcohol abuse was coded as 1 or more indicators of alcohol abuse vs. no indication of alcohol abuse. Similarly, the variable for depression was coded as 1 or more indicators of alcohol abuse vs. no indication of depression vs. no indication.

### Mortality

All-cause mortality was assessed in the nationwide Cause of Death Registry between baseline and 2014 and the follow-up time ended at the date of death, or 31 December 2014, whichever came first.

### Statistical analysis

Cox proportional hazards models with age as the underlying time scale were used to analyse the data. All variables met the proportional hazards assumption. Initially, we estimated hazard ratios (HR) and 95% confidence intervals (CI) for all-cause mortality according to hostility, unemployment, and the joint effect of the two. Level of education and cohabitation status, depression, and alcohol abuse were included in the models and considered as possible confounding factors. By using age as the underlying time scale, we ensured thorough adjustments for age.

All analyses were conducted using SAS version 9.3. The ‘proc phreg’ procedure was used for survival analysis. The synergy index (SI) was used to assess deviation from additivity, which indicates a causal interaction where some cases of the outcome would not have occurred if it had not been for the presence of both hostility and unemployment. The SI calculates the ratio between the combined effect and individual effects of the variables. SI can go from 0 to infinity. SI = 1 means no interaction or exactly additivity, whereas SI > 1 implies positive interaction and SI < 1 implies negative interaction. In this study, SI > 1 is interpreted as evidence in favour of the psychosocial vulnerability model [33]. We followed the guidelines provided by Anderson et al. to calculate regression coefficients and covariance matrix, which was used as an input to calculate the synergy index and 95% CI in excel [33].

## Results

During 14 years of follow-up, 264 men and 201 women died. Table 1 shows the distribution of hostility, employment, cohabitation status, education, age, alcohol abuse, and depression by vital status. More unemployed men and women died compared to those employed. Also, more hostile men and women died compared to those not hostile.

**Table 1:** Hostility, employment, and covariates by vital status Dec. 31, 2014: 8426 Danish men and women from the Danish Longitudinal Study on Work, Unemployment, and Health

Men Women				
	Alive n and row percentage	Dead n and row percentage	Alive n and row percentage	Dead n and row percentage
*Occupation*				
Employed	3217 (94.7)	179 (5.3)	3668 (96.2)	143 (3.8)
Unemployed	386 (82.0)	85 (18.0)	690 (92.2)	58 (7.8)
*Hostility*				
Not hostile	2321 (94.8)	127 (5.2)	3298 (96.1)	139 (3.9)
Hostile	1282 (80.3)	137 (9.7)	1060 (94.4)	63 (5.6)
*Cohabitation*				
Live with partner	2870 (94.9)	153 (5.1)	3398 (96.1)	139 (3.9)
Do not live with partner	733 (86.8)	111 (13.2)	960 (93.9)	62 (6.1)
*Education*				
Secondary education or more	3187 (93.2)	231 (6.8)	3664 (95.8)	159 (4.2)
Primary education	416 (92.7)	33 (7.3)	694 (94.3)	42 (5.7)
*Age*				
< 40 years	114 (92.7)	9 (7.3)	288 (97.6)	7 (2.4)
41 years	1152 (96.6)	40 (3.4)	1326 (97.4)	36 (2.6)
42–50 years	630 (92.8)	49 (7.2)	936 (96.4)	35 (3.6)
51 years	1186 (93.0)	89 (7.0)	1185 (94.9)	63 (5.1)
52+ years	521 (87.7)	77 (12.9)	624 (91.2)	60 (8.8)
*Alcohol abuse*				
No	3464 (94.0)	221 (6.0)	4291 (95.7)	195 (4.3)
Yes	139 (76.4)	43 (23.6)	67 (91.8)	6 (8.2)
*Depression*				
No	3426 (93.7)	232 (6.3)	3989 (96.0)	168 (4.0)
Yes	177 (84.7)	32 (15.3)	369 (91.8)	33 (8.2)

After adjustment for covariates, hostility was associated with higher mortality risk in men, HR 1.47 (95% CI: 1.09–21.99), but not in women, HR 1.17 (95% CI: 0.79–1.74) (Tables 2 and 3). Unemployment was also associated with a higher risk of premature death in both men, HR 2.32 (95% CI: 1.46–3.70) and women, HR 1.78 (95% CI: 1.16–2.73). Men who were *both* hostile and unemployed were at higher risk of premature death, HR 3.19 (95% CI: 2.22–4.69) compared to employed and non-hostile men (Table 2). It is similarly, for women, with a HR 1.98 (95% CI: 1.24–3.12) associated with the joint effect of being hostile and unemployed (Table 3). The synergy index was 1.22 (95% CI: 0.69–2.15) for men and 1.17 (95% CI: 0.76–1.81) for women. Hence, the results did not indicate any synergistic effects between unemployment and hostility.

**Table 2:** Separate and joint effect of hostility and unemployment on all-cause mortality among Danish middle-aged men, *n* = 3867

Separate and joint effects	Alive (n and row percentage)	Dead (n and row percentage)	Model 1^a^ HR 95% CI	Model 2^b^ HR 95% CI
Employed, not hostile (non- exposed)	2174 (95.5)	103 (4.5)	1 (reference)	1 (reference)
Employed, hostile (separate effect)	1043 (93.1)	76 (6.9)	1.59 (CI 1.18–2.15)	1.47 (1.09–1.99)
Unemployed, not hostile (separate effect)	147 (85.6)	24 (14.4)	3.31 (CI 2.12–5.17)	2.32 (1.46–3.7)
Unemployed, hostile (joint effect)	239 (78.9)	61 (21.1)	5.07 (CI 3.67–7.01)	3.19 (2.22–4.69)

^a^Model 1 unadjusted model

^b^Model 2 adjusted education, cohabitation, alcohol abuse and depression

**Table 3:** Separate and joint effect of hostility and unemployment on all-cause mortality among middle-aged Danish women, *n* = 4559

Separate and joint effects	Alive (n and row percentage)	Dead (n and row percentage)	Model 1^a^ HR 95% CI	Model 2^b^ HR 95% CI
Employed, not hostile (non-exposed)	2908 (96.5)	107(3.5)	1 (reference)	1 (reference)
Employed, hostile (separate effect)	760 (95.5)	36 (4.5)	1.25 (CI 0.85–1.84)	1.17 (0.79–1.74)
Unemployed, not hostile (separate effect)	390 (92.7)	31 (7.3)	2.01 (CI 1.32–3.05)	1.78 (1.16–2.73)
Unemployed, hostile (joint effect)	300 (91.6)	27 (8.4)	2.34 (CI 1.51–3.62)	1.97 (1.24–3.12)

^a^Model 1 unadjusted model

^b^Model 2 adjusted education, cohabitation, alcohol abuse and depression

## Discussion

We found that hostility in men and unemployment in both men and women were associated with all-cause mortality. We found no synergistic effects to support the psychosocial vulnerability hypothesis. This suggests that no special vulnerability stems from being exposed to both.

Few studies have addressed the psychosocial vulnerability model in relation to hostility and unemployment. Kivimäki et al. found no interaction between hostility and unemployment on self-rated health among women, but interaction was found among men, that is, hostility accentuated the effect of unemployment on self-rated health in men [11]. Another study by the same first author found an increased vulnerability in hostile women to the stressor of organisational downsizing, job insecurity, and stressful psychosocial work environment in relation to sickness absence. This vulnerability was not observed in men [27]. These studies, however, differ from the present study with regards to the outcome as well as the concept of interaction. Using the concept of causal interaction, other studies have found support for additional deaths due to exposure to both unemployment and being unmarried [34].

Hostility was an independent risk factor in men. It has previously been shown that hostile persons are less likely to engage in social interaction and one of the proposed mechanisms underlying a health consequence of hostility is a lower stress-buffering potential from interpersonal support [11, 25]. Thus, hostility may reduce the health benefits of receiving social support. For example, Holt-Lundstad et al. showed that not only was high hostility associated with heightened systolic and diastolic blood pressure during self-disclosure of stressful events, but those high in hostility also perceived their friends to be less friendly and, consequently, they seemed to benefit less from social support received during stressful periods [25].

In both men and women, unemployment was related to premature mortality. Unemployment represents a severe economically stressful condition, and coping strategies are considered a potential modifier of the impact of unemployment on health and well-being [35]. Thus, inadequate coping among unemployed individuals may set these persons at greater risk of the deleterious health impact of unemployment [36].

### Methodological considerations

Using self-reported psychosocial exposures and subjective health outcomes in observational studies has been called into question [37, 38], which points to the issue of reverse causation via the mechanism that perceived poor health also generates hostility [39]. We consider the register-based outcome measure a major strength in this study, as mortality rules out concerns of reverse causation. Furthermore, to reduce confounding by poor health, we excluded all individuals not working due to illness the year before baseline. Most confounders were also measured using register data, which means that information is complete. However, there are also limitations. Alcohol abuse and depression are detectable only in registers if the individual had been either hospitalised with a main or contributory diagnosis indicating these or had purchased prescription medicines to treat depression or alcohol dependence. These drugs are not sold over the counter. Hence, all treated individuals are in the registers, but some cases of alcohol abuse may never be treated and represent a source of unmeasured confounding (see Additional file 1). Hostility was measured once, just before the start of follow-up. However, hostility is considered to be stable from early adulthood [40]. Hostility in early life is likely to influence several confounding factors throughout the life course such as depression and cohabitation (see DAG in Additional file 2). Unemployment was also measured only once. It is likely that, for example, unemployment and alcohol abuse affect each other sequentially over the life course. We adjusted for alcohol abuse and depression which occurred before the time of the survey. In this way, these factors may be considered confounding factors. However, we realise that this does not capture the complexity of associations between covariates and exposures. For simplicity, this was displayed only in the diagram using T1 to indicate time before survey and T2 to indicate time at survey or after survey. Other health behaviours may be on the causal pathway between hostility and unemployment and mortality, for instance, smoking status, which in contrast to alcohol abuse, is unlikely to affect employment. The relationship between hostility and neuroticism has been shown as possibly two simultaneous dimensions of a disease-prone personality [39, 41] and is thus confounding due to other personality characteristics that may have influenced the relationship between hostility and mortality. Unfortunately, we had no data on other relevant personality factors available in our dataset.

The study population was restricted to individuals eligible for the workforce. Consequently, we cannot generalise our findings regarding psychosocial vulnerability to individuals who are not part of the workforce. As shown in Additional file 1, a relatively large number had missing information on employment. In a sensitivity analysis, we found that those with missing information on employment more often had a low educational level or missing information on both and that their risk of mortality during follow-up was higher than that of the employed but lower than the mortality of the unemployed and sick (data not shown). We did not study causes of death, but we think that the study does contribute to the knowledge of how unemployment interacts with hostility in a broad population exposed to unemployment.

Our theoretical point of departure was the psychosocial vulnerability model and an assumption that hostility is a stable trait in the adult population, but other models of the relations between hostility, social conditions, and health have been suggested, among these the social context model. According to this model, adverse social conditions including unemployment may antecede not only health problems but also hostility [11]. The micro-sociological approach introduced by Thomas Scheff may be of some help in understanding how this causation works. According to Scheff, the individual experiences the social world in emotionally loaded categories which have bodily correlates such as pride, shame, anger, and distrust. The way these emotions are experienced is not uniform throughout the social structure [42]. Further longitudinal studies are needed to investigate relations between hostility, unemployment, and other social factors.

To our knowledge, this is the first study focusing on the joint exposure to unemployment and hostility on the risk of premature death. We found that both hostility (in men only) and unemployment were associated with higher mortality risk, and individuals who were *both* unemployed and hostile were at markedly higher risk of premature death. This may be due to inadequate coping among hostile, unemployed individuals, but our results did not lend support to the psychosocial vulnerability hypothesis. We did not find indication for causal interaction, which implies that, while those exposed to both hostility and unemployment represent a group with high mortality risk, which could benefit from health intervention, the reduced mortality risk from such intervention is not enhanced by synergistic effects.

## Conclusions

Both hostility and unemployment are risk factors for premature death, especially in men, and those exposed to both unemployment and hostility were at markedly high risk of premature mortality. However, this study did not support the hypothesis that the deleterious health effect of the combination of unemployment and hostility exceeds their individual effects.

## Additional files

Additional file 1: Flow chart. The file shows the flowchart of selection of study participants. (DOCX 44 kb)

Additional file 2: DAG and measured variables. The file shows the DAG used for variable selection and an timescale of measurement of included variables. (DOCX 86 kb)

## Abbreviations

CHD: Coronary heart disease
CI: Confidence intervals
CVD: Cardiovascular disease
HR: Hazard ratio
SI: Synergy index
