# Supplementary material for: The joint effect of unemployment and cynical hostility on all-cause mortality: results from a prospective cohort study
Source: BMC Public Health. 2019 Mar 12;19:293. doi: 10.1186/s12889-019-6622-7 (PMC6417173; doi:10.1186/s12889-019-6622-7)
Supplement: Supplementary file 1 — Flow chart. The file shows the flowchart of selection of study participants. (DOCX 44 kb) [file 12889_2019_6622_MOESM1_ESM.docx]

**Flow Chart selection of study population**

Invited to participate in survey 37-56-year-old individuals who had been unemployed at least 70% of the time (marginalized) (N=4,145)

Invited to participate in survey. Random sample of 40 and 50 years in 1999 (N = 11,082)

Missing information on

Hostility 223

Education 100

Total 307

Eligble population

8,733

No register linkage 5

Assesed for inclusion criteria

Included:

7,430 employed at time of survey +1303 unemployed at time of survey

Total 9,870

Did not meet inclusion criteria

Not part of the work force

- Due to illness 486
- Housewifes etc. 276
- Unknown occupation 375

Answered survey Total 9,875

40 and 50 years old 69% =7,588

Marginalized 57% =2,287

9,875

Non responders 3,494

Non responders 1,858

Study population

8,426
