# Supplementary material for: The joint effect of unemployment and cynical hostility on all-cause mortality: results from a prospective cohort study
Source: BMC Public Health. 2019 Mar 12;19:293. doi: 10.1186/s12889-019-6622-7 (PMC6417173; doi:10.1186/s12889-019-6622-7)
Supplement: Supplementary file 2 — DAG and measured variables. The file shows the DAG used for variable selection and an timescale of measurement of included variables. (DOCX 86 kb) [file 12889_2019_6622_MOESM2_ESM.docx]

DAG


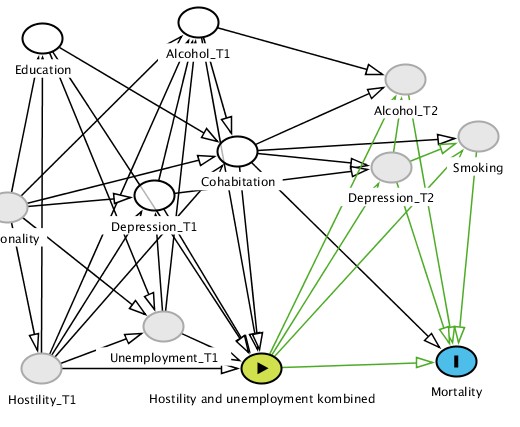


Measured variables

Survey:

Employment status

Hostility

Education

Population registry:

Cohabitation

Hospitalizations related to alcohol abuse and depression 1980-1994 Somatic wards only, 1994-1999 somatic and psychiatric wards. Medications from 1995-1999

Causes of Death registry:

All-cause mortality

1980 1995 1999 2000 2014
